# Prior CT imaging history for patients who undergo PAN CT for acute traumatic injury

**DOI:** 10.7717/peerj.963

**Published:** 2015-06-02

**Authors:** Jeremy Kenter, Osbert Blow, Scott P. Krall, Albert Gest, Cynthia Smith, Peter B. Richman

**Affiliations:** 1 Texas A&M/CHRISTUS Spohn Emergency Medicine Residency, Corpus Christi, TX, USA; 2 Department of Acute Care Surgery, Trauma & Surgical Critical Care, CHRISTUS Spohn Hospital Corpus Christi–Memorial, Corpus Christi, TX, USA

**Keywords:** CT, Trauma, Radiation risk

## Abstract

**Objective.** A single PAN scan may provide more radiation to a patient than is felt to be safe within a one-year period. Our objective was to determine how many patients admitted to the trauma service following a PAN scan had prior CT imaging within our six-hospital system.

**Methods.** We performed a secondary analysis of a prospectively collected trauma registry. The study was based at a level-two trauma center and five affiliated hospitals, which comprise 70.6% of all Emergency Department visits within a twelve county region of southern Texas. Electronic medical records were reviewed dating from the point of trauma evaluation back to December 5, 2005 to determine evidence of prior CT imaging.

**Results.** There were 867 patients were admitted to the trauma service between January 1, 2012 and December 31, 2012. 460 (53%) received a PAN scan and were included in the study group. The mean age of the study group was 37.7 ± 1.54 years old, 24.8% were female, and the mean ISS score was 13.4 ± 1.07. The most common mechanism of injury was motor vehicle collision (47%). 65 (14%; 95% CI [11–18]%) of the patients had at least one prior CT. The most common prior studies performed were: CT head (29%; 19–42%), CT Face (29%; 19–42%) and CT Abdomen and Pelvis (18%; 11–30%).

**Conclusion.** Within our trauma registry, 14% of patients had prior CT imaging within our hospital system before their traumatic event and PAN scan.

## Introduction

Over the past 20 years, computed tomography (CT) has emerged as the imaging modality of choice to evaluate patients for a wide range of pathology (American College of Radiology Appropriateness Criteria, 2012). Consistent with this viewpoint, investigators have identified numerous conditions for which CT appears to enhance diagnostic accuracy in the acute setting. For example, emergency physicians who utilize CT to evaluate patients with abdominal pain appear to significantly reduce the need for surgery (Rosen et al., 2000).

With such benefits in mind, it is not surprising CT use has grown exponentially over the past fifteen years. Investigators reviewing the National Hospital Ambulatory Medical Care Survey observed the utilization of CT expanded 11 times faster than the rate of emergency department visits from 1996 through 2007. In 1996, approximately 3.2 percent of emergency patients received a CT scan. By 2007, the number had risen to almost 14 percent (Kocher et al., 2011).

One area for which there has been significant expansion in CT utilization is for the evaluation of trauma patients. From 1998 to 2007 there was a national 3-fold increase in the use of CT scans in the ER for injury-related conditions (Korley, Pham & Kirsch, 2010). From a diagnostic perspective, such practice appears well supported by findings within the surgical literature over the past decade. Investigators have reported as high as 74% unexpected findings when a PAN scan is utilized in patients where multisystem injury was not anticipated, and the potential to change treatment in 33% of patients (Self & Blake, 2003; Deunk et al., 2007; Deunk et al., 2009).

Despite its apparent utility, the use of PAN CT in acute trauma remains controversial. Recent reports from the emergency medicine literature suggest the possibility of physician judgment guiding focused imaging. While there has been a 3-fold increase in utilization of CT scans for traumatic injury over a decade period, performing these scans has not significantly increased the overall identification rate of life-threatening conditions (Korley, Pham & Kirsch, 2010; Gupta & Schriger, 2011). Of primary concern, the modality presents serious long-term risks of cancer causing morbidity and mortality. A single PAN scan provides significantly more radiation exposure than a conventional x-ray, and at a dose in excess of which is felt to be safe within a one-year period, by the International Symposium on the System of Radiological Protection (20 mSv) (ICRP, 2007). Several published analyses suggest PAN scans could directly result in cancers as often as 1 in 380 and cause 12.5 additional cancer deaths in 10,000 patients (Tien, Tremblay & Rizoli, 2007; Brenner & Elliston, 2001).

As radiation risk increases with higher doses and repeated exposure, patients suffer a higher likelihood of harm/malignancy from a PAN CT if she/he has had one or more earlier CT studies (ICRP, 2007). Despite the apparent importance of prior imaging history for acute setting patients, there is a paucity of research on this topic, and, particularly, with respect to trauma patients. One study, which utilized a questionnaire, found only 14.5% of medical practitioners discuss the risks of radiation exposure, with the patient, prior to CT imaging (Zwank, Leow & Anderson, 2013). Our current study seeks to address a gap in the literature regarding our current understanding of prior imaging history for ED patients. Specifically, we conducted a secondary analysis of a prospectively collected trauma registry and review of a 6-hospital electronic imaging database to test the hypothesis that a significant number of patients who receive a PAN scan for trauma at our institution have had prior CT imaging.

## Materials and Methods

### Study design

This was a secondary analysis of data from a prospectively collected trauma registry followed by a review of corresponding electronic radiological records to evaluate the prevalence of prior imaging within our 6-hospital system.

### Setting

The study was conducted at Christus Spohn Hospital/Corpus Christi—Memorial and five affiliated hospitals. Spohn Memorial is a major teaching affiliate of Texas A&M medical school, a level-two trauma center, and serves an inner-city population. The annual Emergency Department (ED) census is 45,000 patients. The six affiliated hospitals comprise 192,073 annual ED visits, which is 70.6% of all Emergency Department visits within our twelve-county region of southern Texas. The Christus Spohn Institutional Review Board approved the study prior to the initiation of data collection (IRB #13-021), and, due to the retrospective nature of the study/chart review, informed consent was waived.

### Population

The study included all trauma registry patients who received a PAN scan during the period from January 1, 2012 through December 31, 2012. For inclusion into the trauma registry, the patient must undergo a traumatic event and be admitted to the hospital. We used a system-wide electronic medical record and electronic radiology files from our six affiliated hospitals to review the PAN scans and find evidence of prior CT imaging for all patients in the study group dating back to December 5, 2005.

### Statistical analysis

Patient data was recorded on a standardized data collection form and then entered into Excel for Windows (Microsoft Corporation, Redmond, Washington, USA). Subsequently, data was transported into SPSS software (IBM Corporation, Armonk, New York, USA) for statistical analysis. Continuous data is presented as means ± standard deviations and analyzed by *t*-tests; categorical data as frequency of occurrence and analyzed by chi-square. In addition, we calculated 95% CIs and odds ratios. Our primary outcome parameter was the percentage of patients in the trauma registry who were determined to have had a prior CT before their traumatic event. Secondary outcome parameters included identifying which types of CT scans patients with prior imaging history had received.

## Results

There were 867 patients admitted to the trauma service between January 1, 2012 and December 31, 2012. 460 (53%) received a PAN scan and were included in our study group (Table 1). The mean age of the study group was 37.7 ± 1.54 years old, 24.8% were female, and the mean ISS score was 13.4 ± 1.07. The ISS (injury severity score) of patients were observed as follows: ISS <9, 36.5% (32.2–41.0%), ISS 9–16: 27.6% (23.7–31.9%) and ISS >16: 35.9% (31.6–40.3%). The mechanisms of injury included motor vehicle collision (47%), motorcycle collision (13.3%), fall from height (10.5%), and pedestrian struck by vehicle (10%).

**Table 1:** Study group characteristics.

Category	Study group information (*N* = 460)
Mean age	37.7 (sd = 16.8)
Mean ISS score	13.4 (sd = 11.7)
Female gender	114 (24.8%)
Male gender	346 (75.2%)
Motor vehicle collision	216 (47%)
Motorcycle collision	61 (13.3%)
Fall from height	48 (10.5%)
Pedestrian struck by a vehicle	46 (10%)

65 (14%; 95% CI = 11–18%) of the patients had at least one prior CT imaging study. The most common prior studies performed were: CT head (48; 35–58%) which is 2mSV, CT face (30%; 21–38%) which is 1 mSv, and CT abdomen and pelvis (20%; 12–31%) which is 14 mSv. The estimated radiation exposure from these studies are: 2 mSv (millisieverts) 1 mSv, and 14 mSv respectively. (Mettler et al., 2008) Of those with prior imaging, 34% had one previous scan, 30% had two previous scans, 30% had 3–6 scans, and 6% had 7 or more previous scans. One patient had 9 previous CT scans consisting of 5 previous abdominal/pelvis CTs, 2 chest CTs and 2 head CTs. We also examined prior imaging history for young adult patients (age ≤ 35 years; see Table 2) and found a similar prevalence of patients that had prior imaging (38.7%; 24–56%).

**Table 2:** Prior imaging in young adults. Prior CT imaging in adults <35 years of age (*N* = 243).

Mean age:	24.4(SD = 5.6)
Female:	60 (24.7%)
Mean ISS score:	12.8(SD = 11.8)
Mechanism injury:	
MVC	137 (56.4%)
Motorcycle	25 (10.3%)
Fall from height	15 (7.2%)
Pedestrian struck	19 (7.8%)
Received previous CT scans	*n* = 31
1 Prior CT scan:	12 (38.7%; 24–56%)
2 prior CT scans:	10 (32.3%; 18–50%)
3–6 Prior scans	7 (22.6%; 11–40%)
≥7 Prior scans	1 (3.2%; 0–17%)
Common prior studies	
CT head	11 (35.5%; 21–35%)
CT face	10 (32.3%; 18–50%)
CT abdomen pelvis	9 (29.0%; 16–47%)

Table 3 summarizes several subgroup analyses that were performed to compare respective characteristics of patients that had a history of prior imaging. In terms of demographic features, there were no significant differences in terms of the percentage of males who had prior imaging vs. percentage of females who had prior imaging [13.9% vs 14.9%; OR 0.91 (0.50–0.78); *p* = 0.92]. Likewise, we found that white and non-white victims of trauma were similar with respect to previous imaging exposure [12.2% vs. 15.1%; OR =.68 (0.38–1.16) *p* = 0.19]. There was a trend toward older adult trauma patients (age > 55 years) having a higher prevalence of prior imaging versus other age groups; however, this difference was not statistically different (18.4% vs. 13.1%; OR 1.5; .77–2.8; *p* = 0.32). Finally, in terms of injury severity, we did not find that those with more significant injuries were more likely to have had prior CT studies. The average ISS was 12.3 for those with previous CT scans and 13.6 for the group with no previous scans (*p* = 0.40).

**Table 3:** Characteristics of patients with prior imaging.

Subject	Prior imaging	No-prior imaging	OR (95% CI)	*P*-value
% male patients vs % female patients	13.8% 14.9%	86.2% 85.1%	0.91 (0.50–0.78)	0.92
% age < 55 vs % age > 55	13.1% 18.4%	86.9% 81.6%	1.5 (.77–2.8)	0.32
% ISS < 9 vs % ISS > 9	14.3% 27.7%	85.7% 72.3%	1.1 (0.64–1.9)	0.84
% Whites vs % Non-Whites	12.2% 15.1%	87.8% 84.9%	0.68 (0.38–1.16)	0.19

## Discussion

The PAN CT scan is frequently utilized by physicians in the acute trauma setting based on concerns for occult injury, where mechanisms suggest high risk to body organs despite an absence of supporting examination findings. The study typically consists of a non-contrast CT of the Head and Cervical Spine, with an IV contrast CT scan of the chest, abdomen and pelvis. The amount of radiation exposure during this series of CT scans varies from institution to institution, but on average it delivers 22–30 mSv (millisieverts) providing an unusually large radiation dose to patients (Deunk et al., 2009).

Based on current knowledge of radiation exposure risk, investigators estimate that a 37-year-old male has a 1 in 477 chance of cancer in his life as a direct result of receiving a PAN scan alone (Mettler et al., 2008; National Research Council, 2006). Increased utilization of the PAN scan is evident in most hospital systems. One study found an 8% increase in the number trauma patients receiving over 20 mSv of radiation (the recommended threshold yearly dose) after their institution implemented a PAN CT scan protocol (Asha et al., 2012).

Such risks must be weighed against evidence supporting the utility of the imaging study in the surgical literature. For example, Deunk et al. evaluated 106 consecutive blunt trauma patients retrospectively who received a PAN scan to assess the frequency of unexpected findings. An unexpected finding was defined as a positive traumatic injury identified on CT despite negative physical exam, FAST exam, and chest and pelvis x-rays respectively. Of note, 74% of the patients in the study had at least one unexpected finding on their CT scan and 49% of patients had a change in their treatment plan as a consequence of these findings (Deunk et al., 2007).

In a larger series retrospective series, Self and Blake studied 457 trauma patients who had a closed head injury and underwent a PAN scan (CT brain, cervical spine, chest, abdomen and pelvis). Similar to the Deunk et al. methods, if the patient had a no indications of injury prior to the PAN scan (normal physical exam, normal plain films and normal FAST scan), yet had any traumatic abnormality on the CT scan, it was deemed an unexpected finding. Within this database, Self and Blake reported unexpected findings in as high as 38% of cases with changes in management occurring in 26% based on the additional CT images that were obtained (Self & Blake, 2003).

While the PAN CT appears to provide diagnostic benefit, its widespread use based on mechanism alone in the absence of clinically suggestive findings remains controversial. The use of this modality has been questioned from several standpoints beyond the concerns for radiation exposure previously noted. First, the study is expensive with charges to the patient running as high as $17,000 by some accounts and as much as $14,165 in our institution (Gupta & Schriger, 2011).

Second, it is unclear that growing use of CT for trauma has improved the diagnostic yield for life threatening conditions to a degree that warrants this utilization trend. Korley et al., performed a cross-sectional analysis of the National Hospital Ambulatory Medical Care Survey from 1998–2007 and found a 250% relative increase in the use of CT imaging during trauma during that timeframe. However, there was only a small concomitant increase in the detection of life threatening conditions from 1.7% to 2.0% (Korley, Pham & Kirsch, 2010).

Further contributing to the controversy, within the emergency medicine literature, Gupta et al., recently reported physician judgment as a reliable tool to identify low risk patients who would benefit from selective imaging (Korley, Pham & Kirsch, 2010). In this prospective investigation, the authors evaluated 701 trauma patients who underwent a PAN scan. During study encounters, emergency physicians and surgeons were asked in advance to document those parts of the PAN scan they believed would show an abnormality. The authors revealed that If the emergency physicians selectively ordered imaging according to clinical impression/examination, patients would have been exposed to 56% fewer CT scans. With respect to the CT scans felt to be unwarranted, 10% showed an abnormal finding, yet, only 0.3% of those required a critical action. Thus, strictly using emergency physician judgment as a test within the investigation, the negative likelihood ratio of a CT scan resulting in a critical action was 0.05 (Gupta & Schriger, 2011).

Both acknowledged by the Gupta et al. study authors and our current investigation partners, respectively, emergency physicians and trauma surgeons have different comfort levels in terms of defining clinically significant CT findings and acceptable miss rates for actionable injuries. Trauma surgeons consistently express a preference for broad use of PAN scan with resultant lower levels of unrecognized injuries as compared to emergency physicians who seemingly favor selective imaging and might tolerate a higher false negative rate from acting on clinical impression alone. While we don’t expect this controversy to be settled without extensive additional research, we believe that both specialties could agree that in selected lower risk trauma patients there is an opportunity to assess prior imaging exposure and to discuss the risk:benefit profile with patients prior to PAN scanning i.e., an opportunity to empower patients to participate in decisions that balance risk of radiation exposure long-term versus short-term risks of missed significant injury.

Supporting this viewpoint, we present results here that generally confirm our pre-study hypothesis/concern that a significant number of patients admitted to the trauma service following PAN scan had past CT imaging within our six-hospital system antecedent to their acute injury. Within the 460 patient study group, 65 patients (14%; 95% CI [11–18]%) had at least one prior CT imaging study. This number represents nearly 1 in 7 patients admitted to our trauma service. Furthermore, for those with prior imaging, 43 (66%; 53–77%) had more than one imaging study done previously.

Unfortunately, there is little evidence that physicians in the acute setting discuss radiation risk with their patients to any significant extent. Zwank et al. surveyed 200 stable emergency department patients undergoing CT scan about their awareness of radiation risks from CT scans and also inquired as to whether or not their medical provider discussed the risks of radiation exposure that context. They found 25% of patients were aware a CT scan can increase one’s overall lifetime risk of cancer, but only 14.5% of medical providers discussed the risk of radiation prior to the patient receiving a CT scan (Zwank, Leow & Anderson, 2013).

Anecdotally, and more specific to the setting of trauma, we have not seen prior CT imaging history routinely taken by physicians as a component of initial patient history at any institution in our collective experiences. Further, our Medline search did not reveal prior studies investigating this particular area of concern. With federal regulatory bodies (i.e., FDA, CMS) gradually heading toward cooperative analysis and enforcement of standards to reduce patient exposure to radiation from medical imaging, it would seemingly make sense for clinicians at point of care to proactively address the issue through patient education and participation in imaging decision making (US Food and Drug Administration, 2010). Furthermore, as trauma is an unpredictable event in a patient’s future, our study also serves to remind physicians to be selective in their use of imaging modalities with ionizing radiation for elective concerns when alternatives such as MRI and ultrasound may suffice.

## Limitations and Future Directions

Our study has several limitations warranting discussion, particularly with respect to the potential to underestimate the prevalence and frequency of prior CT imaging exposure for trauma patients. For example, our patients could have undergone imaging at other non-affiliated area and/or distant hospitals during the look back period of 7 years. We expect the regional scope of our 6-hospital system limited this possibility, as over 70% of all ED visits within a broad geographic region are represented within this system. Similarly, we also likely underestimated prior imaging history since we were unable to review radiology records dating prior to 2005. Especially in younger patients, remote history of radiation exposure remains relevant to their long-term risk of malignancy. While a fully prospective study might have allowed for surveying individual patients about prior imaging history, such a method might have introduced recollection bias. Likewise, it does not seem easily feasible to conduct a multicenter study of non-academic, non-affiliated community hospitals to directly review all radiological records such that all centers in the region would be represented.

Importantly, the inclusion criteria limited our study group to only those patients admitted to the hospital after receiving their PAN scan i.e., those patients with identified injuries or persistent concern for unrecognized injury following CT. Undoubtedly, the majority of PAN CTs within this group were unavoidable based on clinical suspicion for serious injury and/or distracting injuries that would prevent the clinician from choosing selective imaging. This limitation specifically excluded a large number of individuals seen the Emergency Department for trauma of lower potential acuity who receive a PAN scan and were discharged home. Future studies should focus on this latter group of lower acuity trauma patients for whom discussion of the risk of radiation exposure long-term vs. benefits of CT to avoid missed acute injury may be more balanced.

In view of the aforementioned limitations, we emphasize that our results provide only a lower limit of certainty as to the prior imaging history of our trauma patients who undergo PAN scan. The prevalence of patients receiving prior CT imaging is certainly higher. However, we believe the 14% prior CT imaging history is alarming even before we take into account the likelihood our method underestimates the risk of prior exposure.

## Conclusions

Within our trauma registry, 14% of patients had prior CT imaging within our hospital system before their traumatic event and PAN scan. As serial CTs incrementally increase the lifetime chance of malignancy, this risk should be weighed against evidence supporting the utility of the Pan CT in the primary evaluation of trauma patients.

## Supplemental Information

10.7717/peerj.963/supp-1 Supplemental Information 1 Prior CT for trauma patients data set
